# Cytoreductive surgery without intra-peritoneal chemotherapy for metachronous colorectal peritoneal metastases

**DOI:** 10.1186/s12957-024-03471-w

**Published:** 2024-07-31

**Authors:** Emi Ota, Yosuke Fukunaga, Toshiki Mukai, Yukiharu Hiyoshi, Tomohiro Yamaguchi, Toshiya Nagasaki, Takashi Akiyoshi

**Affiliations:** Department of Gastroenterological Surgery, Cancer Institute Hospital of the Japanese Foundation for Cancer Research, Tokyo, Japan

**Keywords:** Metachronous peritoneal metastases, Cytoreductive surgery, 5-year relapse-free survival, 5-year overall survival, Colorectal cancer

## Abstract

**Background:**

Cytoreductive surgery and chemotherapy reportedly improve the prognosis of patients with metachronous peritoneal metastases. However, the types of peritoneal metastases indicated for cytoreductive surgery remains unclear. Therefore, we aimed to clarify the category of cases for which cytoreductive surgery would be effective and report the prognosis associated with cytoreductive surgery for metachronous peritoneal metastases.

**Methods:**

This study included 52 consecutive patients who underwent cytoreductive surgery for metachronous peritoneal metastases caused by colorectal cancer between January 2005 and December 2018 and fulfilled the selection criteria. The median follow-up period was 54.9 months. Relapse-free survival was calculated as the time from cytoreductive surgery of metachronous peritoneal metastases to recurrence. Overall survival was defined as the time from cytoreductive surgery of metachronous peritoneal metastases to death or the end of the follow-up period.

**Results:**

The 5-year relapse-free survival rate was 30.0% and the 5-year overall survival rate was 72.3%. None of the patients underwent hyperthermic intraperitoneal chemotherapy. The analysis indicated no potential risk factors for 5-year relapse-free survival. However, for 5-year overall survival, the multivariate analysis revealed that time to diagnosis of metachronous peritoneal metastases of < 2 years after primary surgery (hazard ratio = 4.1, 95% confidence interval = 2.0–8.6, *p* = 0.0002) and number of metachronous peritoneal metastases ≥ 3 (hazard ratio = 9.8, 95% confidence interval = 2.3–42.3, *p* = 0.002) as independent factors associated with a poor prognosis.

**Conclusions:**

Long intervals of more than 2 years after primary surgery and 2 or less metachronous peritoneal metastases were good selection criteria for cytoreductive surgery for metachronous peritoneal metastases from colorectal cancer.

## Background

The treatment for peritoneal metastasis from colorectal cancer was previously considered to be only palliative because of its poor prognosis compared with that of other metastases [1]; however, the management of this disease has significantly changed because of cytoreductive surgery, systemic chemotherapy, and hyperthermic intraperitoneal chemotherapy (HIPEC) [2]. In Japan, HIPEC is performed only in limited institutions, as it is not covered by the national insurance system. In contrast, several studies have demonstrated the importance of cytoreductive surgery with systemic chemotherapy for synchronous peritoneal metastasis [3–5]. Furthermore, previous studies on the survival of patients with peritoneal metastasis have focused on synchronous or both synchronous and metachronous types; however, very few reports have focused exclusively on the metachronous type. The incidence of metachronous peritoneal metastasis among patients with colorectal cancer is reported as 3.5–5.1% [6–8]. Furthermore, metachronous peritoneal metastasis might differ from the synchronous type and need to be considered a separate pathological condition associated with a generally better prognosis than the synchronous type [9, 10]. Nagata et al. reported that right colon cancer, early peritoneal metastasis, high peritoneal carcinomatosis index (PCI) score, and concurrent extraperitoneal or distant metastasis were negative prognostic factors for patients with metachronous peritoneal metastasis despite the administration of chemotherapy and appropriate supportive care [11]. However, surgical intervention, even minimally invasive type, often interrupts chemotherapy, resulting in disease progression. In addition, metachronous peritoneal metastasis is likely to be more invasive than the synchronous type because the surgical intervention for the metachronous type is likely a second surgery and involves other organs. As previously stated, it is important to investigate the selection criteria for cytoreductive surgery. Therefore, in this study, we aimed to elucidate the clinical and pathological characteristics of metachronous peritoneal metastasis and provide suggestions regarding the selection criteria for surgical intervention for this disease.

## Methods

### Patient selection

In this study, the data of 128 consecutive patients who underwent cytoreductive surgery for metachronous peritoneal metastasis of colorectal cancer at the Cancer Institute Hospital of the Japanese Foundation for Cancer Research between January 2005 and December 2018 were retrospectively analyzed. Notably, HIPEC was not performed during this period at the hospital. Inclusion criteria were cases with metachronous peritoneal metastasis following primary operation for primary colorectal cancer which had been diagnosed with adenocarcinoma in pathology. Location of the colorectal cancer included appendix, colon, and rectum but not anus. Additionally, cases with other site distant metastasis other than peritoneum were included. The criteria for cytoreductive surgery were as follows: (1) cytoreductive surgery alone, without systemic chemotherapy, was likely chosen for patients with a single peritoneal metastasis that could be easily resected, and (2) systemic preoperative chemotherapy was recommended before cytoreductive surgery for: (i) patients with short intervals to recurrence (within 1 year); (ii) those requiring resection of additional organs involved with the peritoneal metastasis (such as the rectum, uterus, and ovaries), (iii) those with a history of recurrent disease and resection at other sites; (iv) patients with ≥ 2 peritoneal metastases; and (v) patients with distant metachronous metastases, excluding peritoneal types. Cytoreductive surgery was indicated for these patients if they showed stable disease status or partial response following an extended period (6–12 months) of systemic chemotherapy. Furthermore, cytoreductive surgery was considered for patients whose disease status remained stable and for whom chemotherapy had been discontinued at the discretion of the medical oncologist due to severe adverse events or patient compliance issues. The following patients were excluded from this study: (1) those who underwent R2 resection for primary cancer; (2) those who underwent endoscopic submucosal resection for primary cancer; (3) those who underwent R2 resection for metachronous peritoneal metastasis; and (4) those who had only ovarian metastasis. This exclusion criteria were based on the TNM classification of malignant tumors by the Union for International Cancer Control (UICC), 7th edition [12], which classifies ovarian metastasis as distant metastasis ‘M1 (OVA)’ and specifies no peritoneal metastasis ‘P’. The prospective colorectal database was used to collect information regarding patient characteristics, perioperative assessments, and operative characteristics. Primary tumor stage was based on the TNM classification for malignant tumors by the UICC, 7th edition. The follow-up data were extracted from medical records. We were unable to collect all data for KRAS and BRAF mutations and microsatellite instability (MSI) because the technology to do these molecular tests was not available in the early stages of the study. This study was approved by the Institutional Review Board of the Cancer Institute Hospital (protocol no. 2018–1109).

### Diagnosis of metachronous peritoneal metastasis

Metachronous peritoneal metastasis was diagnosed using multiple radiological images (such as computed tomography, magnetic resonance imaging, and positron emission tomography). The final number of metastases was counted and recorded during cytoreductive surgery. If two peritoneal metastases were observed in one organ, they were counted as two metastases. In Japan classically, the rule of ‘Japanese Classification of Colorectal, Appendiceal and Anal Carcinoma’, in which peritoneal metastasis is classified as 0–3 according to the degree, is routinely used for practical treatment of this disease. For example, P1: peritoneal metastasis located near the primary lesion, P2: few metastases in the distant peritoneum, P3: multiple metastases in the distant peritoneum. This study was retrospective so as to use all data recorded according to this rule. Therefore, we did not use PCI to define the extent of peritoneal metastasis in this manuscript. The time to diagnosis was calculated as the interval between the date of the primary surgery and the date of detection of the metastasis. When the time to diagnosis was over 3 months, peritoneal metastasis was defined as metachronous [13].

### Cytoreductive surgery

Basically, only the resection of peritoneal metastasis was performed. For omental metastasis, although most surgeons involved in cytoreductive surgery of colorectal cancer origin perform complete greater omentectomy, in our study partial omentectomy was performed in omental metastatic disease. If the other organs required resection while resecting peritoneal metastasis, multiorgan resection was also performed (such as colorectal resection, total hysterectomy and bilateral salpingo-oophorectomy, and small bowel resection). Salpingo-oophorectomy was not performed for cases with ovarian metastasis alone stated above in the exclusion criterion section.

### Statistical analysis

Data were analyzed using the EZR software package version 3.0 (Saitama Medical Center, Jichi Medical University, Saitama, Japan). The rates of relapse-free survival (RFS) and overall survival (OS) since the resection of the metachronous peritoneal metastasis were calculated using the Kaplan–Meier method, and the factors affecting RFS and OS were analyzed using univariate and multivariate analyses. The log-rank test was used in the univariate analysis, and Cox’s proportional hazard model was used in the multivariate analysis. OS was calculated as the time from cytoreductive surgery to death or the end of the follow-up period. RFS was calculated as the time from cytoreductive surgery to recurrence. The data differences between the groups were considered statistically significant at *p* values < 0.05. This time-to-event analysis provided hazard ratios (HRs) with 95% confidence intervals (CIs).

## Results

### Patient characteristics

**Fig. 1:**
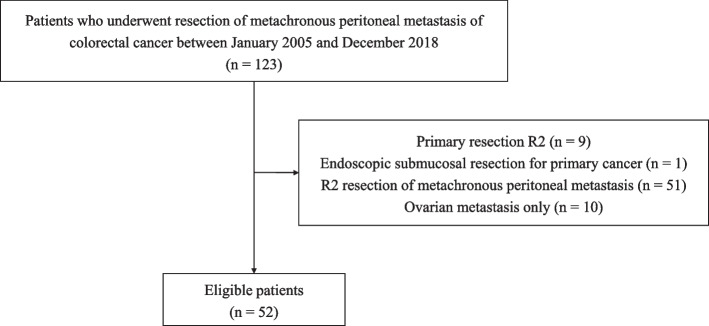
Flowchart of patient selection

Finally, the data of 52 patients were analyzed in this study (Fig. 1). The demographic data of the patients are presented in Table 1. The mean age was 66.5 years, and 36 (69.2%) patients in the cohort were female. A total of 41 patients (78.85%) were diagnosed with primary colon cancer (sigmoid colon cancer was the most common, n = 22, 42.31%) and 11 patients (21.15%) were diagnosed with primary rectal cancer. The pathology of most primary tumors was moderately differentiated adenocarcinoma (n = 43, 82.69%). The depth of the primary tumor was pT3 in 20 patients (38.5%) and pT4 in 30 patients (57.7%), including pT4a in 27 patients (51.9%) and pT4b in three patients (5.8%). Furthermore, lymph node metastasis was positive in 39 patients (75.0%) at the time of primary cancer surgery. Forty-four patients (84.6%) had metachronous peritoneal metastasis alone; by contrast, eight patients (15.4%) had other organ metastases. The locations of distant metastases were the liver (n = 3, 5.77%), lymph nodes (n = 2, 3.85%) and ovaries (n = 3, 5.77%). Furthermore, intra-operative lavage cytology was positive in five patients (9.62%). Forty-five patients (86.54%) had two or fewer peritoneal metastases, and seven (13.46%) had more than three. The resection margin was microscopically negative in 43 patients (82.7%) but positive in nine. The median follow-up period (from the initial diagnosis to the end of data collection) was 54.9 (range, 8.9–162.2) months.

**Table 1:** Characteristics of the 52 cases of metachronous peritoneal metastasis of colorectal cancer

	All (*n* = 52)
Age, median (range), years	66.5 (37–82)
Sex	
Male	16 (30.77%)
Female	36 (69.23%)
BMI, median (range), kg/m^2^	22.9 (18.2–30.86)
Primary cancer	
Appendix cancer	3 (5.77%)
Cecal cancer	10 (19.23%)
Ascending colon cancer	2 (3.85%)
Transverse colon cancer	4 (7.69%)
Sigmoid colon cancer	22 (42.31%)
Rectal cancer	11 (21.15%)
Pathological diagnosis	
Well-differentiated adenocarcinoma	12 (23.08%)
Moderately differentiated adenocarcinoma	31 (59.62%)
Poorly differentiated adenocarcinoma	5 (9.62%)
Mucinous adenocarcinoma	1 (1.92%)
pT	
T1	1 (1.92%)
T2	1 (1.92%)
T3	20 (38.5%)
T4	30 (57.7%)
pN	
N0	13 (25%)
N1	19 (36.54%)
N2	20 (38.46%)
pM1 (primary surgery) (*n* = 6)	
Liver	3 (5.77%)
Pulmonary	1 (1.92%)
Spleen	1 (1.92%)
Lymph node	1 (1.92%)
Distant metastases other than metachronous peritoneal metastasis (metachronous peritoneal metastasis + distant metastasis) (n = 8)	
Liver	3 (5.77%)
Lymph node	2 (3.85%)
Ovary	3 (5.77%)
Number of resections of metachronous peritoneal metastases	
1	40 (76.92%)
2	5 (9.62%)
3	3 (5.77%)
4	1 (1.92%)
5	1 (1.92%)
6	2 (3.85%)
Intra-operative lavage cytology	
Negative	47 (90.38%)
Positive	5 (7.62%)
Operative time, median (range), min	143 (29–668)
Blood loss, median (range), mL	80 (0–2650)
Time to diagnosis of metachronous peritoneal metastasis, median (range), m	27.65 (4.87–150.23)
Follow-up duration, median (range), m	54.9 (8.9–162.2)

*BMI* body mass index

Values represent numbers (percentages) unless indicated otherwise

### Time to diagnosis of metachronous peritoneal metastasis

The median time to diagnosis was 27.65 (range 4.9–150.2) months for metachronous peritoneal metastasis, 27.65 months for colon cancer, and 28.87 months for rectal cancer. Almost half (n = 25, 48.08%) of the metachronous peritoneal metastases were detected within the first 2 years of the primary surgery, and nearly all patients were diagnosed within 5 years (n = 45, 86.54%).

### Risk factors for 5-year RFS

**Fig. 2:**
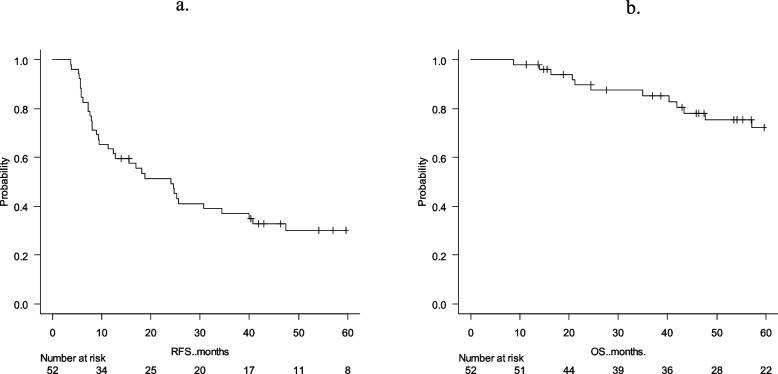
Survival curves based on resection of metachronous peritoneal metastasis. **a**) 5-year relapse-free survival; **b**) 5-year overall survival

The survival curves of 5-year RFS are shown in Fig. 2a. The median RFS of all cases was 24.0 months. The results of the univariate and multivariate analyses of 5-year RFS are shown in Table 2. The univariate analysis showed no risk factors for 5-year RFS. There were no significant differences between pT1, pT2, pT3 and pT4; pN0, pN1 and pN2; and R0 and R1 resection, with or without distant metastasis, the time to diagnosis of metachronous peritoneal metastasis from primary surgery, intra-operative lavage cytology results, and number of resections for peritoneal metastases.

**Table 2:** Univariate and multivariate analyses of 5-year relapse-free survival in 52 cases of resection of metachronous peritoneal metastasis

Variable	All (*n* = 52)		Univariate analysis	Multivariate analysis	
	No. of patients	5-year RFS	*p*-value	HR (95% CI)	*p*-value
Age, years			0.452		
< 70	40 (76.92%)	31.7%			
≥ 70	12 (23.08%)	NA			
Sex			0.716		
Male	16 (30.77%)	34.7%			
Female	36 (1.92%)	27.8%			
Primary site			0.642		
Colon cancer	41 (78.85%)	25.2%			
Rectal cancer	11 (21.15%)	45.5%			
CEA (ng/ mL)			0.623		
≤ 5	27 (51.92%)	29.6%			
> 5	25 (48.08%)	30.6%			
CA19-9 (U/ mL)			0.858		
≤ 37	43 (82.69%)	31.3%			
> 37	9 (17.31%)	27.8%			
pR0, 1 (primary surgery)			0.79		
R0	48 (84.75%)	30.5%			
R1	4 (7.69%)	25.0%			
pT			0.165		
T1, 2, 3	22 (42.31%)	44.3%			
T4	30 (57.69%)	19.4%			
pN			0.487		
N0, 1	31 (59.62%)	31.1%			
N2	21 (40.38%)	27.9%			
pM			0.22		
M0	41 (78.85%)	33.2%			
M1	11 (21.15%)	18.2%			
ly			0.18		
ly0	6 (11.54%)	66.7%			
ly1, 2, 3	46 (88.46%)	25.9%			
v			0.943		
v0	13 (25.0%)	30.8%			
v1, 2, 3	39 (75.0%)	29.3%			
Pathological diagnosis			0.622		
Well, moderately differentiated adenocarcinoma	46 (88.46%)	28.6%			
Poorly differentiated, mucinous adenocarcinoma	6 (11.54%)	NA			
RAS			0.628		
Wild	18 (34.6%)	15.3%			
Mutant	19 (36.5%)	23.0%			
BRAF			–		
Wild	41 (78.8%)	21.7%			
Mutant	0	–			
MSI			–		
MSS	8 (15.4%)	37.5%			
MSI-high	0	–			
Distant metastases other than metachronous peritoneal metastasis			0.616		
No	46 (88.46%)	31.9%			
Yes	6 (11.54%)	16.7%			
Time to diagnosis of metachronous peritoneal metastasis			0.675	0.8626 (0.4458–1.669)	0.6606
< 2 years	27 (51.92%)	28.6%			
≥ 2 years	25 (48.08%)	32.0%		0.8489 (0.2987–2.413)	
Number of resections of metachronous peritoneal metastases			0.78		0.7586
< 3	45 (86.54%)	29.2%			
≥ 3	7 (13.46%)	NA			
Intraperitoneal lavage cytology			0.948		
Negative	47 (90.38%)	29.2%			
Positive	5 (9.62%)	40.0%			
pR0, 1 (resection of metachronous peritoneal metastasis)			0.539		
R0	43 (82.69%)	32.0%			
R1	9 (17.31%)	22.2%			
Preoperative systemic chemotherapy			0.454		
No	26 (50.0%)	23.9%			
Yes	26 (50.0%)	35.0%			
Postoperative chemotherapy			0.288		
No	29 (55.77%)	23.6%			
Yes	23 (44.23%)	39.6%			

*HR* hazard ratio, *CI* confidence interval

### Risk factors for 5-year OS

**Fig. 3:**
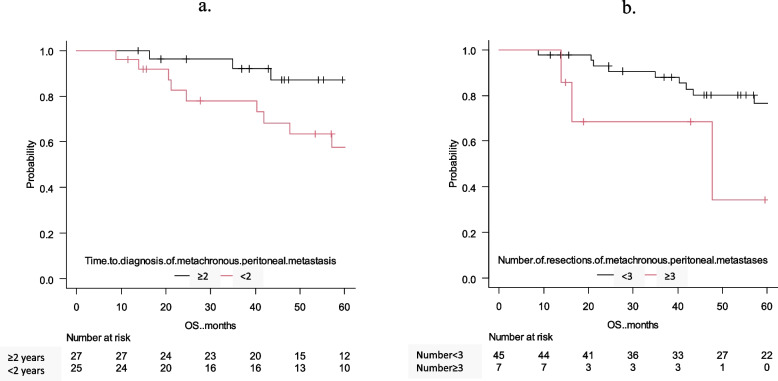
Survival curves based on resection of metachronous peritoneal metastasis **a**) 5-year overall survival curves based on diagnosis time of metachronous peritoneal metastasis; **b**) 5-year overall survival curves based on number of resections of metachronous peritoneal metastases

**Fig. 4:**
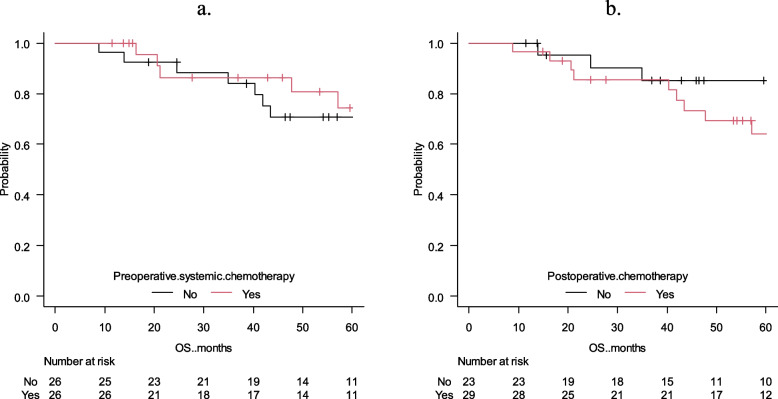
Survival curves based on resection of metachronous peritoneal metastasis. **a**) 5-year overall survival curves based on preoperative systemic chemotherapy; **b**) 5-year overall survival curves based on postoperative chemotherapy

The survival curves of 5-year OS are shown in Fig. 2b. The 5-year survival in all cases was 72.3%. The results of the univariate and multivariate analyses of the 5-year OS are shown in Table 3. The univariate analysis revealed a significantly shorter OS in patients for whom the time to diagnosis of metachronous peritoneal metastasis was < 2 years after primary surgery (*p* = 0.011) and the number of metachronous peritoneal metastases was ≥ 3 (*p* = 0.038). Furthermore, multivariate analysis was performed for significant factors (*p* < 0.05) found in the univariate analysis. The time to diagnosis of metachronous peritoneal metastasis after primary surgery of < 2 years (HR = 4.1, 95% CI = 2.0–8.6, *p* = 0.0002) and number of metachronous peritoneal metastases ≥ 3 (HR = 9.8, 95% CI = 2.3–42.3, *p* = 0.002) were extracted as independent factors associated with poor prognosis. Figure 3 shows the survival curves of the 5-year OS with time to diagnosis of metachronous peritoneal metastasis and the number of resections of metachronous peritoneal metastasis. Figure 4 shows no difference in 5-year OS with or without preoperative or postoperative systemic chemotherapy. Twenty-six (50.0%) patients received preoperative chemotherapy and 23 (44.23%) patients received postoperative chemotherapy. FOLFOX, XELOX or FOLFIRI were used; targeted therapies such as bevacizumab, panitumumab and cetuximab were used in 18 (34.6%) patients. No patient received immunotherapy. Chemotherapies were introduced 8 (range 2–58) cycles.

**Table 3:** Univariate and multivariate analyses of 5-year overall survival in 52 cases of resection of metachronous peritoneal metastasis

Variable			Univariate analysis	Multivariate analysis	
	No. of patients	5-year OS	*p*-value	HR (95% CI)	*p*-value
Age, years			0.257		
< 70	40 (76.92%)	76.8%			
≥ 70	12 (23.08%)	51.9%			
Sex			0.244		
Male	16 (30.77%)	84.0%			
Female	36 (1.92%)	67.0%			
Primary site			0.748		
Colon cancer	41 (78.85%)	71.3%			
Rectal cancer	11 (21.15%)	75.0%			
CEA (ng/ mL)			0.738		
≤ 5	27 (51.92%)	81.4%			
> 5	25 (48.08%)	63.3%			
CA19-9 (U/ mL)			0.681		
≤ 37	43 (82.69%)	73.9%			
> 37	9 (17.31%)	64.8%			
pR0, 1 (primary surgery)			0.745		
R0	48 (84.75%)	69.8%			
R1	4 (7.69%)	100%			
pT			0.708		
T1, 2, 3	22 (42.31%)	71.3%			
T4	30 (57.69%)	73.5%			
pN			0.797		
N0, 1	31 (59.62%)	71.3%			
N2	21 (40.38%)	73.3%			
pM			0.162		
M0	41 (78.85%)	73.8%			
M1	11 (21.15%)	67.3%			
ly			0.163		
ly0	6 (11.54%)	100%			
ly1, 2, 3	46 (88.46%)	60.1%			
v			0.0965		
v0	13 (25.0%)	90.0%			
v1, 2, 3	39 (75.0%)	67.2%			
Pathological diagnosis			0.819		
Well, moderately differentiated adenocarcinoma	46 (88.46%)	71.1%			
Poorly differentiated, mucinous adenocarcinoma	6 (11.54%)	83.3%			
RAS			0.968		
Wild	18 (34.6%)	81.7%			
Mutant	19 (36.5%)	74.7%			
BRAF			–		
Wild	41 (78.8%)	75.6%			
Mutant	0	–			
MSI			–		
MSS	8 (15.4%)	100%			
MSI-high	0	–			
Distant metastases other than metachronous peritoneal metastasis			0.44		
No	46 (88.46%)	71.0%			
Yes	6 (11.54%)	83.3%			
Time to diagnosis of metachronous peritoneal metastasis			0.0109	4.19 (1.343–13.07)	0.01361
< 2 years	27 (51.92%)	87.1%			
≥ 2 years	25 (48.08%)	57.7%			
Number of resections of metachronous peritoneal metastasis			0.0383	4.33 (1.140–16.45)	0.03134
< 3	45 (86.54%)	76.6%			
≥ 3	7 (13.46%)	NA			
Intraperitoneal lavage cytology			0.821		
Negative	47 (90.38%)	74.7%			
Positive	5 (9.62%)	50.0%			
pR0, 1 (resection of metachronous peritoneal metastasis)			0.448		
R0	43 (82.69%)	72.4%			
R1	9 (17.31%)	71.4%			
Preoperative systemic chemotherapy			0.711		
No	26 (50.0%)	74.4%			
Yes	26 (50.0%)	70.7%			
Postoperative chemotherapy			0.156		
No	29 (55.77%)	63.9%			
Yes	23 (44.23%)	85.2%			

HR, hazard ratio; CI, confidence interval

## Discussion

Metachronous peritoneal metastasis is an important prognostic factor that cannot be ignored after primary surgery for colorectal cancer. In this study, we analyzed the data of consecutive patients who underwent cytoreductive surgery for metachronous peritoneal metastasis caused by colorectal cancer and found that cytoreductive surgery was effective for patients with the time to diagnosis of metachronous peritoneal metastasis of ≥ 2 years after primary surgery and < 3 metachronous peritoneal metastases. Although there are several supportive reports of combined HIPEC and cytoreductive surgery [14–18], most recent randomized controlled trials of COLOPEC and PRODIGE 7 revealed no additional effect of HIPEC on cytoreductive surgery alone for peritoneal metastasis of colorectal cancer [19, 20]. A multi-center cohort study from PSOGI, including Japanese institutions investigating the characteristics of long-term survivors of this disease, reported no differences between cytoreductive surgery alone and in combination with HIPEC [21, 22]. Cytoreductive surgery with or without HIPEC has been shown to be associated with long-term survival [23, 24]. Additionally, in Japan, HIPEC is not covered by the national insurance system, this procedure hence, is rarely performed. In this study, chemotherapy and cytoreductive surgery without HIPEC provided a favorable prognosis. To the best of our knowledge, this is the largest study that focused on cytoreductive surgery without HIPEC for metachronous peritoneal metastasis.

Some studies have reported the prognosis of patients with metachronous peritoneal metastasis as better than that of the synchronous type [9, 10]; however, others have reported similar prognosis for both types [11, 18, 25–29]. Additionally, the prognosis of patients with metachronous peritoneal metastasis was significantly better in patients who underwent cytoreductive surgery or combined cytoreductive surgery and chemotherapy (median survival time 60.0 months) than that of patients with synchronous type (median survival time 52.6 months) [11]. In our series, the median RFS of 24.0 months, 5-year RFS of 30.0%, and 5-year OS of 72.3% seemed to be favorable compared with the findings of previous reports.

Regarding the interval to the diagnosis of metachronous peritoneal metastasis after primary cancer surgery, several studies reported a median interval of approximately 14–18 months, with 83% of the cases being detected within 3 years of initial diagnosis and 98% being diagnosed within 5 years [6, 16, 30]. Similarly, in this study, the median interval was 27.65 months, with 48.08% (*n* = 25) of the cases detected within 2 years, 65.38% (*n* = 34) within 3 years, and 86.54% (*n* = 45) within 5 years. Patients with an interval to diagnosis longer than 2 years had better prognosis than those with a shorter interval within 2 years. This trend has also been reported by Nagata et al. [11].

Considering prognostic factors, pT4a is regarded as the most important independent risk factor. Nagata et al. reported that metachronous peritoneal metastasis was observed in 16% of the patients with pT4 in stage I–III colorectal cancer and that pT4 was an independent risk factor for this disease [11]. This trend was also observed in our study, wherein patients with pT4a accounted for the majority of the cohort (57.7%). There was no significant difference in prognosis between patients with and without lymph node metastasis in our study. However, Sugarbaker and Chang reported that peritoneal metastases from colorectal cancer in the absence of lymph node metastases showed a high survival rate [31]. Second, the incidence of a history of distant metastasis (reported in 37–60% of patients with metachronous peritoneal metastasis) and prior liver or lung resection for metastasis have been considered to be associated with poor prognosis [7, 11, 32]. However, contrary reports have been recently published showing the history of distant metastasis as not being a significant factor for prognosis if complete resection was possible [29, 33]. In this study, 15.38% (*n* = 8) of the patients had a history of distant metastasis, but it did not affect the prognosis. Interestingly, this incidence did not differ between patients with R0 and R1 resection for peritoneal metastasis, implying a favorable prognosis even in patients with distant metastasis based on complete macroscopic resection. Additionally, shrinkage of the lesion by combining chemotherapy with cytoreductive surgery can improve the ratio of complete resection.

Cytoreductive surgery for metachronous peritoneal metastasis has often been selected in cases limited to less than two metastases on preoperative imaging in our hospital thus far. Most cases with more than three metachronous peritoneal metastases without systemic chemotherapy before surgery were diagnosed incidentally during surgery despite having less than two metastases on preoperative images. Preoperative systemic chemotherapy was not a prognostic factor related to RFS or OS in the multivariate analysis in our study. In the PRODIGE 7 trial, HIPEC combined with cytoreductive resection and cytoreductive resection alone did not lead to differences in RFS and OS but significantly increased operation time, hospitalization period, and the incidence of neutropenia, thrombopenia, and any complication (grade ≥ 3 adverse events). Additionally, no differences were reported in terms of prognosis between the no chemotherapy and chemotherapy (both postoperative and perioperative systemic chemotherapy) groups. Based on this result, cytoreductive surgery and systemic chemotherapy can provide encouraging survival rates in patients with very limited metachronous peritoneal metastasis (1–2 metastatic nodules). This is equivalent to a PCI score < 6. This is also consistent with the results of the PRODIGE 7 trial, where no difference was observed between HIPEC and no HIPEC groups in patients with PCI score 1–10. Therefore, HIPEC may be not necessary for those with very limited peritoneal metastasis. Depending on the PCI score, different treatment strategies may be needed: HIPEC may be useful for high PCI score, while cytoreductive surgery may be useful for very limited peritoneal metastasis with low PCI score. Although the degree of metachronous peritoneal metastasis has been reported as a major prognostic factor for cytoreductive surgery [34, 35], no reports has mentioned the number of peritoneal metastases. Whereas the PCI score may vary in lesion size depending on how to measure and who measures, counting the “number of metastases” used in our study is simple and fare. It would be able to be widely used and may be an adjunct diagnosis way to PCI. Before cytoreductive surgery, staging laparotomy may also be considered, and if there are more than three metachronous peritoneal metastases, cytoreductive surgery, especially when it would be highly invasive, may need to be avoided. Regarding long-term outcomes, no significant factors influenced RFS; however, the time to diagnosis of metachronous peritoneal metastasis and number of lesions extracted were independent factors related to OS. These results imply that patients with a long-interval of diagnosis for metachronous peritoneal metastasis over 3 years or those with fewer than three localized lesions might likely undergo repeated surgery in case of recurrence after the primary surgery.

The strength of this study is its large sample size investigating metachronous peritoneal metastasis of colorectal cancer. However, several limitations should also be considered when interpreting our results. First, this was a retrospective study at a single institution, and 45 out of 52 patients had one or two peritoneal lesions as only resectable cases were selected, there may have been a bias in case selection. We also need to consider more advanced cases in the future study. Also survival results between patients receiving or not receiving preoperative systemic chemotherapy could be biased by the fact that chemotherapy was given to patients with more advanced disease and/or unfavorable prognostic features. Analogously, the trend for better overall and -free survival seen in patients who had postoperative systemic chemotherapy could be biased by the fact that patients in a better condition after a challenging surgery were more likely to receive systemic chemotherapy. Second, because only surgical cases of metachronous peritoneal metastasis were investigated, prognostic data of cases that could not be selected for cytoreduction were not compared. We could not ascertain whether cytoreduction for this disease contributed to improve long-term outcomes compared with systemic chemotherapy alone by this investigation. Third, although there were reports showing the importance of KRAS and BRAF mutations and MSI for prognosis of cytoreductive surgery [36–40], we could not collect these data in the early stages of the study. Finally, the study spanned over a significant period (2005–2018), during this time advancements in treatments, such as molecularly targeted drugs and care strategies, may have influenced the outcomes. In the future, we would like to include data on biological aspects along with molecular testing and the use of targeted agents or immunotherapy with or without chemotherapy, for further consideration. Forth, PCI might be a better prognostic factor, commonly used worldwide, than the number of peritoneal metastases. Thus, we need further study to convert the number of peritoneal metastases to the PCI score and to discern its relevance in this study. A large-scale randomized controlled study would be desired to elucidate the effects of cytoreduction for this disease.

## Conclusions

Long intervals of more than 2 years after primary surgery and fewer than 3 metachronous peritoneal metastases were good selection criteria for cytoreductive surgery for metachronous peritoneal metastasis of colorectal cancer.

## Data Availability

No datasets were generated or analysed during the current study.

## Abbreviations

CI: Confidence interval
HIPEC: Hyperthermic intraperitoneal chemotherapy
HR: Hazard ratios
MSI: Microsatellite instability
OS: Overall survival
PCI: Peritoneal Carcinomatosis Index
RFS: Relapse-free survival
